# Human bone marrow-mesenchymal stem cells differentiation into brain-like endothelial cells

**DOI:** 10.55730/1300-0152.2786

**Published:** 2025-08-05

**Authors:** Yomna SOLIMAN, Gülin BARAN, Nur MUSTAFAOĞLU

**Affiliations:** 1 Molecular Biology, Genetics, and Bioengineering, Faculty of Engineering and Natural Sciences, Sabancı University, İstanbul, Turkiye; 2 Biochemistry Department, Faculty of Pharmacy, Mansoura University, Mansoura, Egypt; 3 Sabancı University Nanotechnology Research and Application Center, İstanbul, Turkiye

**Keywords:** Brain microvascular endothelial cells (BMECs), mesenchymal stem cells (MSCs), cobalt chloride (CoCl_2_), sodium sulfite (Na_2_SO_3_), retinoic acid (RA)

## Abstract

**Background/Aim:**

Brain microvascular endothelial cells (BMECs), which constitute the blood–brain barrier (BBB), are essential for maintaining central nervous system homeostasis. Like BMECs, multipotent mesenchymal stem cells (MSCs) originate from the mesodermal lineage. Thus, MSCs may serve as a direct and efficient cellular source for BMEC-like differentiation. Notably, differentiation of human induced pluripotent stem cells (hiPSCs) into BMECs typically involves a 2-step protocol: inducing mesodermal commitment followed by endothelial specification. In contrast, direct differentiation from MSCs could bypass the initial mesodermal induction step, offering a streamlined alternative. This study tested a novel strategy for differentiating MSCs into brain-like endothelial cells (BLECs), circumventing the conventional mesodermal induction step.

**Materials and methods:**

Our differentiation protocol integrates developmental cues through the application of hypoxia, retinoic acid (RA), cobalt chloride (CoCl_2_), and—for the first time in this context—sodium sulfite (Na_2_SO_3_) to promote endothelial specification. Various basal media, including IMDM, EGM-2, and Endopan, were tested in combination with B27 supplement or fetal bovine serum (FBS) to optimize differentiation conditions. MSC viability under CoCl_2_ and Na_2_SO_3_ treatment was evaluated using the MTT assay to determine appropriate concentrations. The endothelial functionality of the resulting BLECs was assessed via tube formation assays.

**Results:**

Immunocytochemical analysis confirmed the expression of key BMEC markers, including ZO-1, CD31, and occludin, showing both phenotypic and functional characteristics of brain microvascular endothelium.

**Conclusion:**

This MSC-based differentiation approach provides a robust and physiologically relevant in vitro BBB model with potential applications in studying neurological disease mechanisms and screening therapeutic agents.

## Graphical Abstract

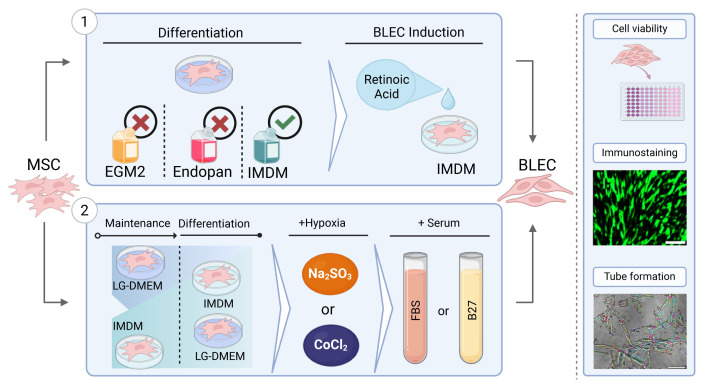

## 1. Introduction

The blood–brain barrier (BBB) consists of brain microvascular endothelial cells (BMECs) lining the blood vessels, supported by the extracellular matrix (ECM), pericytes, and astrocytes, forming a microenvironment essential for BBB function (Cecchelli et al., 2007). Unlike peripheral capillaries, BMECs are characterized by tight junctions that restrict paracellular permeability and necessitate transcytosis for the transport of molecules into the central nervous system (CNS) (Greene and Campbell, 2016). Additionally, BMECs express broad-spectrum efflux pumps that limit the uptake of lipid-soluble compounds into the brain, including numerous drugs (Mahringer et al., 2011; Shawahna et al., 2011). Astrocytes and pericytes provide crucial signals for BMEC differentiation (Abbott and Friedman, 2012; Herland et al., 2016). Collectively, these 3 cell types maintain BBB integrity (Helms et al., 2015; Wolff et al., 2015). During embryogenesis, mesoderm-derived endothelial cells (ECs) form a vascular plexus surrounding the developing neural tube (Risau and Wolburg, 1990; Era et al., 2008). The canonical Wnt signaling pathway is essential for BMEC barrier properties as nascent blood vessels invade the developing CNS (Liebner et al., 2008; Stenman et al., 2008; Paolinelli et al., 2013). Retinoic acid (RA) has also been implicated in BMEC specification, with radial glial cells supplying RA to the CNS during BBB development (Mizee et al., 2013), thereby promoting BBB-specific gene expression and barrier formation (Mizee et al., 2013, 2014; Lippmann et al., 2014a).

In vitro and in vivo BBB models are widely used to study neurovascular function and dysfunction in disease states (Helms et al., 2015; Soliman et al., 2024). However, animal models alone fail to fully recapitulate human BBB physiology and transporter activities, and human primary or immortalized BMEC cultures have inherent limitations (Weksler et al., 2005; Syvänen et al., 2009; Bernas et al., 2010). Consequently, various stem and progenitor cells have been explored to generate BMECs in vitro, including hematopoietic stem cells (Cecchelli et al., 2014), endothelial progenitors (Boyer-Di Ponio et al., 2014), and human pluripotent stem cell (hPSC)-derived ECs cocultured with pericytes, astrocytes, and neurons (Yamamizu et al., 2017) or C6 glioma cells (Minami et al., 2015). Among these, hPSCs have emerged as the leading source for BMEC differentiation, having key BBB properties such as tight junction formation, transporter expression, and polarized efflux transporter activity (Weidenfeller et al., 2007; Lian et al., 2012; Lippmann et al., 2012; Lippmann et al., 2014b; Stebbins et al., 2016; Park et al., 2019; Goldeman et al., 2021). However, the time, cost, and technical expertise required for hPSC differentiation limit their widespread adoption (Hollmann et al., 2017).

BMECs originate from the mesodermal lineage during embryonic development (Pittenger et al., 2019). In line with this, some studies have utilized a hiPSC-derived BMEC differentiation approach that follows the intermediate mesoderm stage (Lu et al., 2021; Patel et al., 2024). Given that mesenchymal stem cells (MSCs) also originate from the mesoderm and have self-renewal and multipotent properties (Qian et al., 2017; Pittenger et al., 2019), they may represent a promising alternative strategy for generating MSC-derived BMECs. MSCs have been differentiated into ECs through coculture with endothelial progenitor cells (Ge et al., 2018), high-density MSC cultures (Whyte et al., 2011), HUVEC lysates (Lozito et al., 2009), chemically fixed EC layers (Joddar et al., 2018), and endothelial ECM coatings (Gong et al., 2017). Coculture methods activate Notch signaling, thereby increasing vascular endothelial growth factor A (VEGF-A) and EC marker expression, cobblestone morphology, and tube formation (Gong et al., 2017; Joddar et al., 2018).

Hypoxia plays a critical role in regulating MSC stemness, differentiation, and proliferation (Sart et al., 2014). From embryonic development to maturity, the hypoxic environment is crucial for cell renewal and repair (Abdollahi et al., 2011). While excessive hypoxia can lead to CNS damage, mild hypoxia, such as that induced in intermittent hypoxia therapy, has neuroprotective effects (Li et al., 2021). Cobalt chloride (CoCl_2_) is widely used to mimic hypoxia in vitro by stabilizing hypoxia inducible factor (HIF)-1α (Kim et al., 2006; Teti et al., 2018; Chen et al., 2019). In this study, we introduce sodium sulfite (Na_2_SO_3_) as another hypoxia-mimicking agent for the differentiation protocols on the mammalian cells for the first time. Na_2_SO_3_ scavenges O_2_ molecules and metal ions, such as Co^2+^, Zn^2+^, and Ni^2+^, upregulating HIF-1α (Collaco et al., 2006; Kaczmarek et al., 2009) and its downstream targets erythropoietin, B-cell lymphoma 2, and VEGF-A.

In this study, we developed a novel MSC-to-BLEC differentiation protocol using different key factors, including RA, hypoxia-inducing agents (CoCl_2_ and Na_2_SO_3_), and Iscove’s modified Dulbecco medium (IMDM). We compared serum-free and serum-supplemented media. The differentiated BLECs expressed key BMEC markers (ZO-1, occludin, and CD31) and formed tubes on Matrigel, highlighting their functional capabilities.

## 2. Materials and methods

### 2.1. Preparation of MSCs expansion medium

Low-glucose Dulbecco’s modified eagle medium (LG-DMEM) and IMDM were prepared using LG-DMEM and IMDM basal medium, respectively, supplemented with 10% hi-fetal bovine serum (FBS), 1% pen/strep, and 1% 200 mM L-Glu (only for LG-DMEM). The media were sterile filtered and stored at 4 °C.

### 2.2. Preparation of endothelial differentiation media

IMDM-D, LG-DMEM-D, EGM-2-D, and Endopan-D refer to IMDM, LG-DMEM, EGM-2, and Endopan basal media, respectively, supplemented with 2% FBS, vascular endothelial growth factor (VEGF), basic fibroblast growth factor (bFGF), insulin-like growth factor (IGF), epidermal growth factor (EGF), ascorbic acid, heparin, and, where applicable, hydrocortisone and gentamycin/amphotericin (G/A) (specifically for EGM-2-D and Endopan-D). All media were sterile filtered and stored at 4 °C. Detailed compositions are provided in supplementary Table 1.

In summary, Endopan-D and EGM-2-D were prepared using the manufacturer’s instructions, including their respective growth supplement kits. In contrast, IMDM-D and LG-DMEM-D were prepared following a previously described formulation (Wang et al., 2018), with modifications. Specifically, instead of the original supplement composition, we used growth supplements from the Lonza BulletKit (Lonza, Visp, Switzerland, catalog number: CC-4176) to enhance endothelial differentiation.

### 2.3. MSCs culture

MSCs were purchased from ATCC (Manassas, VA, USA, catalog number: PCS-500-012), and passages 10–23 were utilized during the study. MSCs were expanded in complete LG-DMEM at varying seeding densities according to the type of culture container and the experiment. Upon reaching 80–90% confluency, cells were washed with DPBS −/−, trypsinized using trypsin/EDTA (Pan-Biotech GmbH, Aidenbach, Germany), and replated into new culture flasks, petri dishes, or well plates (Isolab GmbH, Eschau, Germany).

### 2.4. Differentiation of MSCs into BLECs

All experiments were designed to have 2 replicates of each condition, with MSCs serving as the negative control. MSCs were seeded at 1 × 10^5^, 2 × 10^5^, 3 × 10^5^, and 4 × 10^5^ cells/well in a 6-well plate and 1 × 10^4^, 7.5 × 10^3^, 5 × 10^3^, 4 × 10^3^, and 2 × 10^3^ cells/well in 48-well plates, and cultured for 24 h. Where applicable, cells were preconditioned in 50 μM CoCl_2_ or 4 mM Na_2_SO_3_ in the expansion medium for 24 h to induce hypoxia. The medium was then replaced with endothelial differentiation media (Endopan, EGM-2, IMDM, or LG-DMEM) with or without hypoxia regulators. For RA experiments, differentiation media were supplemented with 1, 3, and 10 μM RA either for 48 or 72 h starting on day 6 or day 12 of differentiation. For the subculture of the differentiated cells, differentiated cells were washed, trypsinized, counted, and replated on noncoated 48-well plates at 2 × 10^3^ cells/well.

### 2.5. Immunofluorescence staining

Upon completing the differentiation protocol, cells were observed under a light microscope (Carl Zeiss Primovert, Oberkochen, Germany 3841016470), washed 3 times with DPBS +/+ (Pan-biotech), and fixed with 4% paraformaldehyde (Merck, Darmstadt, Germany) for 20 min at room temperature in the dark. Cells were then incubated in blocking buffer, consisting of 1.5% bovine serum albumin (Pan-Biotech), 0.01% Tween 20 (Sigma-Aldrich, St Louis, MO, USA) in DPBS +/+, for 1 h at room temperature. Cells were washed 3 times with DPBS +/+, and then incubated overnight at 4 °C with primary antibodies against ZO-1 (ProteinTech, Rosemont, IL, USA, 21773-1-AP, 1:500), CD31 (ProteinTech, 11265-1-AP, 1:500), and occludin (ProteinTech, 27260-1-AP, 1:500). After washing with DPBS +/+ 3 times, cells were incubated with the Cy3 AffiniPure Goat antirabbit IgG (H+L) (Jackson Immuno Research, West Grove, PA, USA, 111-165-003, 1:1000) for 1 h at room temperature in the dark, followed by DAPI staining (Sigma-Aldrich, MBD0015, 1:1000) for 2 min. Cells were washed, mounted in DPBS +/+, and imaged using a Zeiss Vert.A1 fluorescence microscope.

### 2.6. Image quantification using ImageJ

Fluorescence intensity of the immunostained images, with 20× magnification power over 6 analyzed fields, was quantified using ImageJ. Normalized mean fluorescence intensity (NMFI) was determined by selecting specific regions of interest for both test and only secondary Ab images. The MFI was then normalized to cell numbers, averaged, and analyzed in GraphPad Prism version 10.2.3(403). Background fluorescence from secondary antibody images was subtracted from test samples (fNMFI_test_ = iNMFI_test_ − NMFI_only secondary Ab_).

### 2.7. MTT assay

Cells were seeded at a density of 5 × 10^3^ cells/well in LG-DMEM with 10% FBS and cultured for 24 h at 37 °C with 5% CO_2_. Cells were then treated with either CoCl_2_ (0.005–400 μM) or Na_2_SO_3_ (0.025–2000 μM) for 5 to 9 days. Control cells were left untreated. A 50 μL aliquot of MTT reagent (3-(4,5-dimethylthiazol-2-yl)-2,5-diphenyltetrazolium bromide, Sigma-Aldrich, MKBZ5197V) was added into each well, and cells were incubated for 3–4 h. After removing the supernatant, 200 μL of solubilization solution (40% v/v) was added, and plates were shaken for 10 min to dissolve the formazan crystals. Absorbance at 570 nm was measured using a TECAN infinite M200 Pro Tecan plate reader (Männedorf, Switzerland). Experiments were performed in triplicate. Cell viability was plotted using GraphPad Prism, with logarithmic concentration on the x-axis and percentage viability value on the y-axis.

### 2.8. Tube formation assay

Following 9 days of endothelial differentiation, cells were harvested with trypsin and seeded at 1 × 10^4^ cells/well in 96-well plates precoated with 50 μL Matrigel (Corning, Corning, NY, USA). After 24 h, tube formation was visually assessed under phase contrast optics. Total tube and segment lengths were quantified using an ImageJ angiogenesis analyzer plugin.

### 2.9. Statistical analysis

Statistical comparisons were performed using 1-way and 2-way analysis of variance (ANOVA). Data are presented as mean ± standard error of the mean (SEM). Statical significance was set at p < 0.05.

## 3. Results and discussion

There are numerous protocols for differentiating hPSCs into BLECs, utilizing iPSCs, hematopoietic stem cells (HSCs), or ESCs, but none using MSCs. Notably, Qian et al. (2017) differentiated iPSCs into mesodermal lineage cells, which were subsequently induced into BLECs. Building upon these findings, we aimed to differentiate MSCs into BLECs by leveraging protocols designed for iPSCs and MSCs-to-ECs differentiation (Lippmann et al., 2013; Lippmann et al., 2014a; Wilson et al., 2015; Wang et al., 2018; Pong et al., 2020). By integrating key elements from these protocols, we identified crucial factors for the development of an optimized MSC-derived BLEC differentiation strategy.

### 3.1. Morphological characterization of MSCs

Bone marrow-derived MSCs (BM-MSCs) were spindle shaped and had fibroblast-like morphology during passaging and maintenance in LG-DMEM expansion medium (supplementary Figure 1a). As the cells proliferated, they aligned parallel to one another, following a characteristic whirlpool-like growth pattern (supplementary Figure 1b). Increased seeding densities and prolonged culture durations resulted in higher cell death rates and the formation of cellular aggregates (supplementary Figure 1c). CD105 expression was higher in undifferentiated MSCs compared to differentiated cells in IMDM medium (supplementary Figures 1d and 1e).

### 3.2. Influence of medium composition on BM-MSC differentiation

VEGF is a key regulator of endothelial differentiation and vascular development (Ferrara, 2004). VEGF activates multiple signaling pathways involved in EC proliferation and differentiation (Bhakuni et al., 2016). bFGF similarly facilitates MSC-to-EC differentiation (Xu et al., 2013), and previous studies suggest that VEGF and bFGF act synergistically to promote vasculogenesis (Asahara et al., 1995). EGF has also been implicated in EC motility, maturation, and vasculogenesis (Volz et al., 2017). Furthermore, EGF may stimulate angiogenesis independently of VEGF through activation of the PI3K and MAPK signaling pathways (Mehta et al., 2007). Although IGF contributes to EC differentiation, its efficacy is lower than that of VEGF (Nicosia et al., 1994). Additionally, hydrocortisone enhances the endothelial barrier properties (Furihata et al., 2015). Heparins facilitate the binding of many proteins to high-affinity receptors on cells, particularly within the endothelium (Ling et al., 2016). Factors such as VEGF and FGF-2 normally associate with heparan sugars on cell surfaces to form ligand–sugar–receptor complexes that induce proliferative signals (Khorana et al., 2003). Finally, vitamin C (ascorbic acid), prevents endothelial barrier leakage and tightens the endothelial barrier (Ulker et al., 2016).

Given that modifications to culture conditions and differentiation media can influence lineage commitment and differentiation kinetics (Pankratz et al., 2007; Chambers et al., 2009; Lippmann et al., 2014b), we investigated the effects of different media compositions on MSC-to-BLEC differentiation. Specifically, we compared 3 differentiation media: Endopan, IMDM, and EGM-2. We also evaluated the effects of seeding density and differentiation duration on cellular morphology and marker expression.

### 3.3. Optimization of differentiation conditions

Collectively, BM-MSCs were expanded in LG-DMEM until they reached 80–90% confluency, after which the medium was switched to:

1. EGM-2 medium (Pankajakshan et al., 2013) where cells were seeded at similar densities and maintained for 10 days with medium changes every 3 days.
2. IMDM differentiation medium (Wang et al., 2018) where cells were seeded at 5 × 10^3^, 1 × 10^4^, 2 × 10^4^, and 4 × 10^4^ cells/well in a 48-well plate with medium changes daily for 14 days.
3. Complete Endopan differentiation medium where cells were seeded at varying densities (1 × 10^5^, 2 × 10^5^, 3 × 10^5^, and 4 × 10^5^ cells/well in a 6-well plate) and maintained in Endopan for 14 days with medium changes every 2 days.

The differentiated cells had distinct morphological changes, transitioning into short, spindle-shaped structures (Supplementary Figure 2a). At later stages, the cells reached confluency (Supplementary Figure 2b). EGM-2-cultured cells had consistent growth patterns, unlike IMDM-cultured cells that had little discernible growth pattern during differentiation. For Endopan, cellular aggregation was observed by day 9 (Supplementary Figure 2c), prompting a reduction in seeding density to enhance cell viability until day 14. To mitigate this issue, subsequent experiments utilized lower seeding densities. Additionally, immunostaining results showed that Endopan-, IMDM-, and EGM-2-treated cells expressed ZO-1 and CD31 surface markers.

### 3.4. Marker expression analysis

Following the initial optimization, we compared the differentiation efficiency of IMDM, Endopan, and EGM-2 at a standardized seeding density (1 × 10^4^ cells/well in a 48-well plate) at differentiation days 7 and 14 (supplementary Figure 3a). Endopan and IMDM induced the highest expression of ZO-1 and CD31, respectively, on day 7 compared to EGM-2 (supplementary Figure 3b).

Immunofluorescence staining was performed over a time course (2, 5, 8, 11, and 14 days) using 2 seeding densities (5 × 10^3^ and 7.5 × 10^3^ cells/well). ZO-1 expression peaked with EGM-2 at day 8 in the higher seeding density and with IMDM at days 5 and 11 in both the higher and lower seeding densities (supplementary Figure 3c and 3d). CD31 expression was highest in IMDM-treated cells on most days for both seeding densities (supplementary Figure 3c and 3d). These results suggest that day 8 was the optimal time point for differentiation.

### 3.5. Effect of medium change frequency on differentiation efficiency

**Figure 1:**
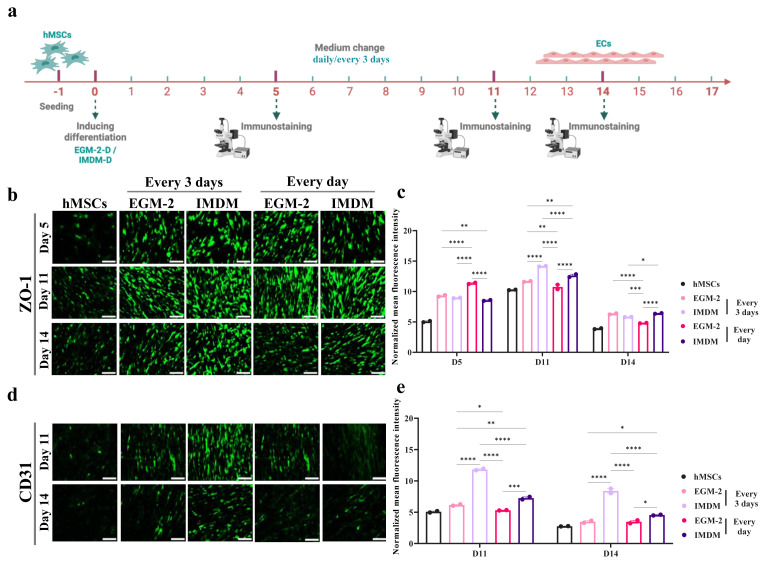
How frequently the medium is changed plays a role in the differentiation. (a) A schematic timeline for the experiment. (b) and (d) ZO-1 and CD-31 expressions, respectively, tested by immunofluorescence staining. (c) and (e) The fluorescence intensity of ZO-1 and CD-31, respectively, quantified in Fiji software and analyzed in GraphPad Prism. *p < 0.05, **p < 0.01, ***p < 0.001, and ****p < 0.0001 from 2-way ANOVA comparisons. All the scale bars are 25 μm.

Since MSCs secrete growth factors and cytokines (Bartaula-Brevik et al., 2017), we investigated whether altering the medium change frequency (daily vs. every 3 days) would influence differentiation efficiency. IMDM and EGM-2 media were tested at a reduced seeding density of 4 × 10^3^ cells/well. ZO-1 expression was higher expression at days 5 and 11 for EGM-2 with daily medium changes and IMDM with medium changes every 3 days, respectively. CD31 expression indicated that IMDM with medium changes every 3 days consistently produced higher marker expression (Figure 1). Thus, IMDM with medium changes every 3 days was selected as the optimal differentiation condition.

In summary, IMDM and EGM-2 had superior differentiation efficiency compared to Endopan. Higher seeding densities promoted marker expression but also led to excessive cell aggregation and detachment. IMDM was ultimately chosen as the preferred differentiation medium, particularly when the medium was changed every 3 days, as it yielded the highest expression of endothelial markers. These findings align with previous reports of efficient MSC-to-EC differentiation in IMDM (Zhang et al., 2008; Chen et al., 2009; Wang et al., 2018).

### 3.6. RA impact o n MSC-to-BLEC differentiation

**Figure 2:**
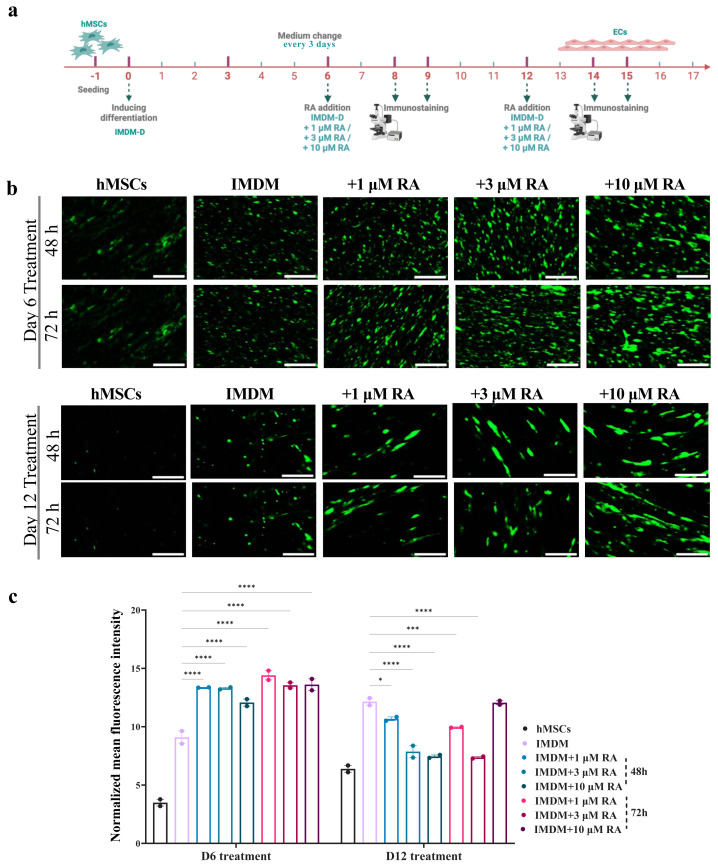
Impact of RA on MSC differentiation into BLECs. (a) A schematic timeline for the experiment where BM-MSCs differentiated in IMDM at 4 × 10^3^ cells/well in 48-well plates for 8, 9, 14, and 15 days with the addition of 1, 3, and 10 μM RA on day 6 and 12 of differentiation. (b) ZO-1 expression tested by immunofluorescence staining. (c) The fluorescence intensity quantified in Fiji software and analyzed in GraphPad Prism. *p < 0.05, ***p < 0.001, and ****p < 0.0001 from 2-way ANOVA comparisons. All the scale bars are 110 μm.

To further optimize the differentiation protocol, we incorporated RA—a key factor commonly used in iPSC-derived BLEC protocols. RA, released by radial glial cells during CNS development, is hypothesized to confer physiological BBB characteristics to immature BMECs (Mizee et al., 2013). Previous studies have shown that RA significantly enhances the passive barrier properties of BMECs throughout differentiation (Lippmann et al., 2014a). To evaluate the effect of RA on barrier integrity, we first tested ZO-1 and CD-31 expressions after 48 h and 72 h of 1 μM of RA addition to IMDM on days 6 and 12 of differentiation. The results proposed increasing the concentration of RA (supplementary Figure 4a and 4b). Therefore, we then tested different concentrations (1 μM, 3 μM, and 10 μM) and assessed ZO-1 expression after 48 and 72 h when RA was added on either day 6 or day 12 of differentiation. The results showed higher expressions at day 6 treatment with no significant difference between all the concentrations; therefore, the chosen condition was 3 μM RA applied on day 6 (Figure 2). Although the increase in expression was lower than anticipated, the addition of RA shortened the differentiation period from 14 days to 8 days. These findings suggest that additional factors may be required to further enhance ZO-1 expression and improve barrier function efficiency.

To confirm our results, we tested the expressions of more markers, including CD-34, occludin, and von Willebrand factor (vWF). vWF generally has higher expression in normal ECs than in brain ECs (Mojiri et al., 2019; Mbagwu and Filgueira, 2020; Hennigs et al., 2021), while occludin is a more specific marker for brain endothelial cells (Mokoena et al., 2025). CD-34 is expressed differentially in different tissues but tends to be expressed more in brain tissues (Haber et al., 2015). Our results showed high CD-34 and very high occludin expression when RA was added (supplementary Figure 5). vWF was expressed at a higher level in the IMDM-only condition (supplementary Figure 5), proving the ability of RA to induce brain-like characteristics in differentiated MSCs.

### 3.7. Chemical hypoxia induces the differentiation of BM-MSCs into BLECs

The BBB initially develops in the embryonic brain under low oxygen (O_2_) conditions (1–8%) before the establishment of the circulatory system (Abdollahi et al., 2011). Endothelial differentiation from various stem cell sources is strongly influenced by oxygen availability (Lee et al., 2012; Kusuma et al., 2014). Based on these observations, we hypothesized that mimicking hypoxic conditions could enhance the differentiation of BM-MSCs into BLECs and potentially stabilize their phenotype. Additionally, the formation of brain microvessels is known to depend on canonical Wnt/β-catenin signaling, with Wnt7a and Wnt7b playing key roles in BBB development in vivo (Daneman et al., 2009; Delsing et al., 2018).

Moreover, previous in vitro and in vivo investigations have shown that the Wnt/β-catenin signaling pathways interact with the HIF-1α signaling pathway (Zhang et al., 2013; Xu et al., 2017). By stabilizing HIF-1α, CoCl_2_ has previously been shown to chemically imitate the consequences of hypoxia (Kim et al., 2006). Compared to normoxic conditions, iPSCs exposed to 100 μM cobalt chloride (CoCl_2_) for 9 days of differentiation had elevated HIF-1α levels (Park et al., 2019). CoCl_2_ was tested over the whole differentiation period of the MSCs-BLECs differentiation protocol.

Given that the safe concentration window of CoCl_2_ is 200 μM (Teti et al., 2018), we initially tested this concentration, along with 150 μM, 100 μM, and 50 μM, by adding them to IMDM throughout the 8-day differentiation period. However, the cells did not survive until the end of differentiation. We then tried to decrease the duration of exposure to CoCl_2_ by adding the higher concentrations to IMDM for 24 h and 48 h. The results showed the maximum time at which the cells would stay vivid and show higher expression was 48 h (supplementary Figure 6a).

**Figure 3:**
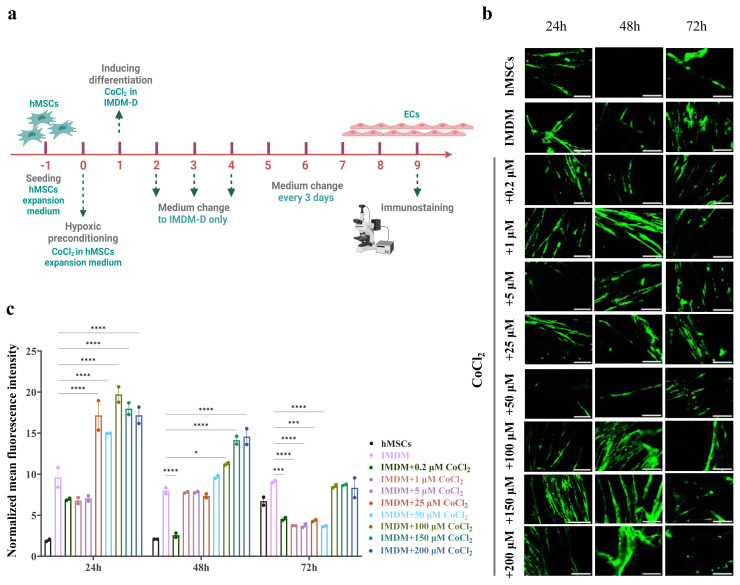
Chemical hypoxia induced by CoCl_2_ affects BM-MSCs differentiation. (a) A schematic timeline of the experiment shows BM-MSCs cultured in the IMDM differentiation medium at 2 × 10^3^ cells/well in 48-well plates for 9 days. (b) ZO-1 expression tested by immunofluorescence staining. (c) The fluorescence intensity quantified in Fiji software and analyzed in GraphPad Prism. *p < 0.05, ***p < 0.001, and ****p < 0.0001 from 2-way ANOVA comparisons. All the scale bars are 110 μm.

Many MSC differentiation protocols utilizing hypoxic conditions incorporate hypoxic preconditioning (HPC), which involves adding CoCl_2_ to the expansion medium 24 h before differentiation induction (Chacko et al., 2010; Muzakkir et al., 2020). HPC upregulates prosurvival and proangiogenic markers (Chacko et al., 2010). Therefore, we applied HPC for 24 h using lower CoCl_2_ concentrations (25 μM, 5 μM, 1 μM, and 0.2 μM), but the cells still did not survive until the end of differentiation. Further reductions in CoCl_2_ concentrations (0.15 μM, 0.1 μM, 0.05 μM, and 0.025 μM) were tested; however, these conditions did not induce significant ZO-1 expression (supplementary Figure 6b). To optimize the protocol, we introduced higher and lower CoCl_2_ concentrations (ranging from 0.2 to 200 μM) into IMDM for 24, 48, and 72 h, respectively, after a previous preconditioning of MSCs with the same concentrations for 24 h. Notably, ZO-1 expression was significantly upregulated under these conditions, particularly at higher CoCl_2_ concentrations and specifically for 24 h and 48 h conditions, while 72 h results were nonsignificant (Figure 3). Based on these findings, which align with our previous findings of maximum vitality at 48 h in differentiation medium with CoCl_2_, we established an optimized protocol in which MSCs undergo 24-h preconditioning with CoCl_2_ in the expansion medium, followed by 24 h of treatment in IMDM differentiation medium.

To determine the optimal concentration of CoCl_2_ that could be both effective and nontoxic over the 9-day differentiation protocol, various concentrations of CoCl_2_ (ranging from 0.5 μM to 400 μM and 0.025 μM to 50 μM) were tested for cellular toxicity using the MTT assay over 5 and 9 days of culture. Among these, 50 μM was the highest concentration that remained nontoxic throughout the 9 days (Supplementary Figures 7a and 7b). In summary, CoCl_2_ can be used at high concentrations for 24 h in the differentiation medium or 50 μM for the 9-day differentiation period.

### 3.8. Optimization of hypoxia-induced differentiation using sodium sulfite

To further explore hypoxia-mediated differentiation, we investigated the effect of Na_2_SO_3_. The hypoxic state induced by Na_2_SO_3_ influences MSC stemness, proliferation, and differentiation (Sart et al., 2014). In *Caenorhabditis elegans* models, 5.5–22 mM Na_2_SO_3_ has been used to simulate hypoxia, with 11 mM effectively creating a hypoxic microenvironment (Bin et al., 2010).

To determine the optimal Na_2_SO_3_ concentration for MSC differentiation, we tested concentrations ranging from 2 mM to 16 mM in IMDM for 24 h. Higher concentrations (8 mM and 16 mM) were highly toxic, resulting in significant cell death, whereas 2 mM and 4 mM were well tolerated. To confirm the noncytotoxicity of Na_2_SO_3_, an MTT assay was conducted using concentrations ranging from 0.025 μM to 4 mM over 5 and 9 days of culture. All tested concentrations were found to be nontoxic (supplementary Figure 7c). Based on these findings, 4 mM Na_2_SO_3_ was selected as the optimal concentration for hypoxia induction.

### 3.9. FBS proves to be superior to B27 in inducing MSC differentiation into BLECs

**Figure 4:**
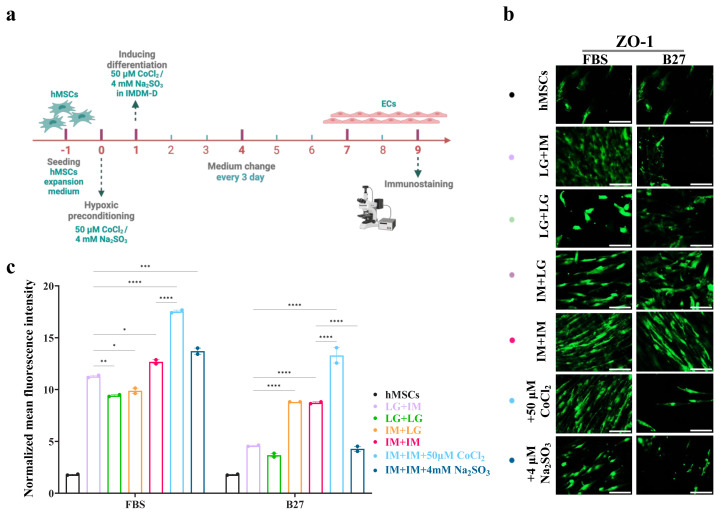
Different mixes of LG-DMEM and IMDM basal media for expansion and differentiation of BM-MSCs. (a) A schematic timeline for the experiment shows BM-MSCs cultured in LG-DMDM or IMDM differentiation media supplemented with either FBS or B27 at 2 × 10^3^ cells/well in 48-well plates for 9 days. (b) ZO-1 expression on day 9 of differentiation. ZO-1 expression tested by immunofluorescence staining. (c) The fluorescence intensity quantified in Fiji software and analyzed in GraphPad Prism. *p < 0.05, **p < 0.01, and ****p < 0.0001 from 2-way ANOVA comparisons. All the scale bars are 100 μm.

In all previous experiments, LG-DMEM was used as the expansion medium, while IMDM was used as the differentiation medium. To assess the impact of different basal media combinations on BLEC differentiation, we systematically varied the expansion and differentiation media, generating 4 conditions: LG-DMEM + IMDM, LG-DMEM + LG-DMEM, IMDM + LG-DMEM, and IMDM + IMDM (Figure 4). Serum-free media have been used in some iPSC differentiation protocols, particularly in neuronal cultures (Hollmann et al., 2017; Qian et al., 2017; Neal et al., 2019). To assess whether serum-free conditions could support MSC differentiation into BLECs, we replaced FBS with the B27 supplement.

**Figure 5:**
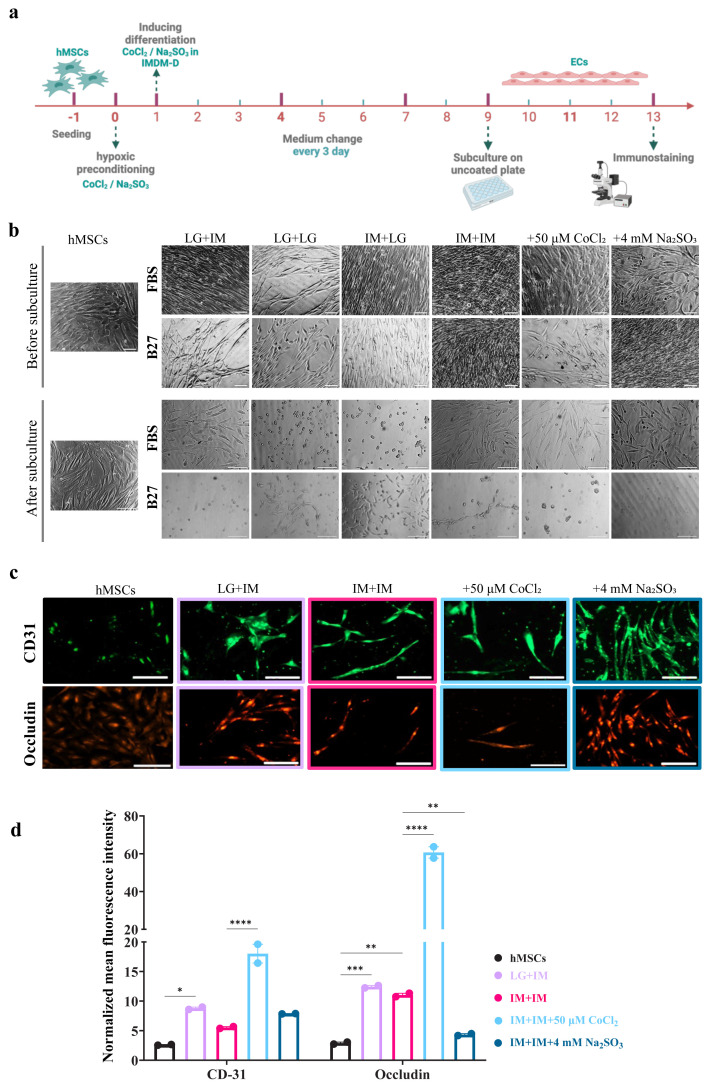
Effect of subculturing the cells on uncoated plates. (a) A schematic timeline of the experiment shows BM-MSCs cultured in LG-DMDM or IMDM differentiation media supplemented with FBS or B27, with a seeding density 2 × 10^3^ cells/well in 48-well plates for 9 days and then subcultured on noncoated plates. (b) Bright-field images of the cells on day 9 of differentiation (before subculture) and after 4 days (after subculture) in the same medium on uncoated plates. (c) CD-31 and occludin expression on day 4 after subculture, tested by immunofluorescence staining. (d) The fluorescence intensity quantified in Fiji software and analyzed in GraphPad Prism. *p < 0.05, **p < 0.01, ***p < 0.001, and ****p < 0.0001 from 2-way ANOVA comparisons. The scale bars of before and after subcultures are 110 μm and 215 μm, respectively. The scale bars of (c) are 235 μm.

ZO-1 expression was significantly higher in IMDM than LG-DMEM (Figure 4). Additionally, cells cultured in LG-DMEM during expansion and differentiation were more rounded (Figure 5), whereas those differentiated in IMDM were more spindle like (Figure 5). Immunostaining experiments further confirmed these findings; ZO-1 expression was higher in cells differentiated in FBS-supplemented media across all conditions. Among these, cells differentiated using the IMDM + IMDM condition had the strongest ZO-1 expression. Notably, the addition of 50 μM CoCl_2_ to the IMDM + IMDM condition resulted in the highest ZO-1 expression observed across all tested conditions (Figure 4). CoCl_2_ treatment resulted in stronger ZO-1 expression localized at the cell periphery, while Na_2_SO_3_ treatment increased expression to a lesser extent (Figure 4). Similarly, in B27-supplemented conditions, the IMDM + IMDM group had the highest ZO-1 expression, followed by the IMDM + LG-DMEM condition. Interestingly, the addition of 4mM Na_2_SO_3_ to IMDM + IMDM did not enhance ZO-1 expression in the B27-supplemented condition, suggesting future studies can focus on increasing the concentration of Na_2_SO_3_. These findings indicate that 4mM Na_2_SO_3_ is not as effective as 50 μM CoCl_2_ in inducing hypoxia and suggest that FBS is more suitable than B27 for MSC differentiation into BLECs.

### 3.10. Subculture of differentiated cells causes differences in attachment to the noncoated plates

To evaluate the attachment properties of differentiated cells, we subcultured them onto new uncoated plates (Figure 5). In general, cells differentiated in the presence of FBS had better attachment after subculturing, with the notable exceptions of the LG-DMEM + LG-DMEM and IMDM + IG-DMEM conditions, where attachment was not observed. Conversely, cells differentiated with the B27 supplement had poor attachment across all conditions. Among the tested groups, only cells from the IMDM + LG-DMEM condition successfully reattached after subculturing. Interestingly, in the LG-DMEM + LG-DMEM condition, a small population of cells was observed despite the lack of attachment in the corresponding FBS condition, suggesting a potential variation in cell behavior under different differentiation environments.

Furthermore, CD31 and occludin expression in the subcultured cells were significantly higher expression in cells treated with CoCl_2_ compared to those in the IMDM + IMDM and LG-DMEM + IMDM conditions, particularly for occludin (Figures 5c and 5d). These findings highlight the critical role of basal media composition and serum supplementation in determining the reattachment potential and endothelial marker expression of differentiated cells. Furthermore, the results prove the strong effect of CoCl_2_ in inducing hypoxia and the brain-like characteristics of differentiated cells.

### 3.11. Tube formation confirms endothelial functionality

**Figure 6:**
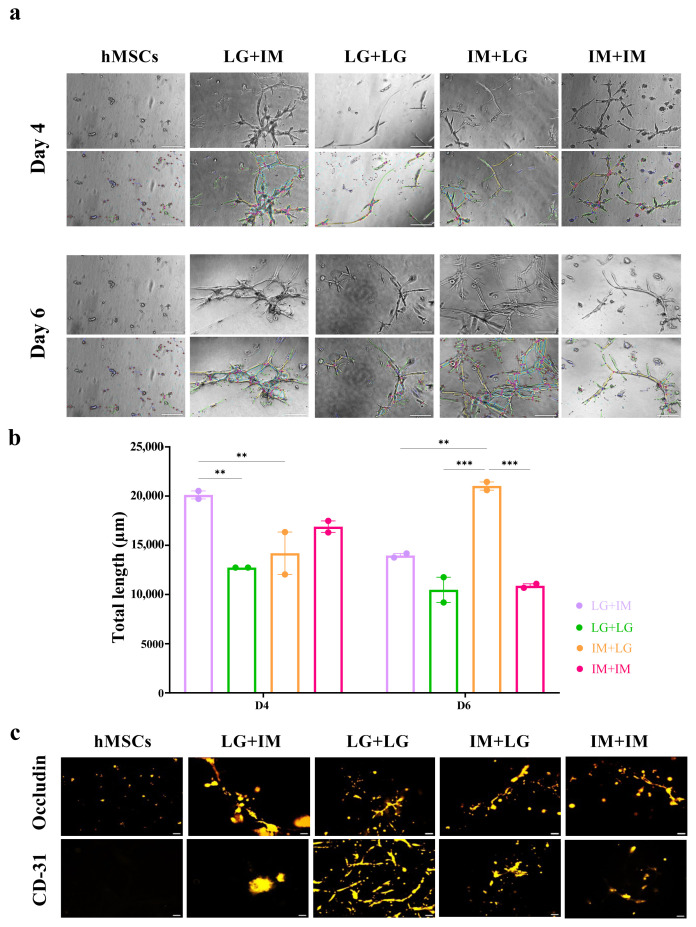
Tube formation assay. BM-MSCs cultured in LG-DMDM or IMDM differentiation media at 2 × 10^3^ cells/well in 48-well plates for 9 days. (a) Phase contrast images of the formed tubes day 4 and 6 on Matrigel and (b) normalized total tube lengths. (c) CD-31 and occludin expressions on the formed tubes tested by immunofluorescence staining. **p < 0.01 and ***p < 0.001 from 2-way ANOVA comparisons. The scale bars of (a) and (c) are 200 μm and 120 μm, respectively.

A defining characteristic of functional ECs is their ability to undergo angiogenesis. Extensive studies have shown that ECs can form vascular networks in vitro (Tancharoen et al., 2017). In an angiogenesis assay using Matrigel, undifferentiated MSCs aggregated and failed to form capillary-like structures, as expected, since they lack the intrinsic ability to generate vascular networks. In contrast, following differentiation into BLECs, vessel-like structures formed (Figure 6a), showing the endothelial functionality of the differentiated cells.

Quantitative analysis of tube formation using ImageJ showed that both IMDM and LG-DMEM differentiation media induced MSC differentiation into functional ECs effectively, leading to the formation of extensive tubular networks (Figure 6b). Furthermore, immunostaining of the formed structures confirmed the expression of endothelial markers CD31 and occludin, indicating the acquisition of endothelial characteristics (Figure 6c). These findings suggest that differentiated BLECs have endothelial functionality, making them promising candidates for therapeutic neovascularization and BBB modeling.

## 4. Conclusion

In conclusion, this study showed that MSCs can differentiate into BLECs effectively and form vessel-like constructs in vitro when cultured in an endothelial-inducing environment. The use of fully defined medium components, along with the application of hypoxia regulators (CoCl_2_ and Na_2_SO_3_) and retinoic acid (RA), significantly enhanced the BLEC phenotype. Our findings highlight the sensitivity of MSC-derived BLECs to both the composition of the extracellular environment and the basal medium used during differentiation and cultivation. We show that the defined medium supports the efficient generation of BLECs. These advancements may improve the accessibility and reproducibility of MSC-derived BLECs, facilitating the development of high-fidelity human in vitro BBB models for various applications. However, given the preliminary nature of these findings, further validation of barrier integrity and functionality using a broader range of MSC sources is needed by incorporating differentiated BLECs into in vitro BBB platforms. These follow-up studies are currently ongoing in our laboratory. Nevertheless, the present study provides a comprehensive understanding of the key factors influencing MSC-to-BLEC differentiation and lays the groundwork for future applications in brain disease modeling and regenerative medicine.

## Supplementary information for

A novel human bone marrow-mesenchymal stem cells differentiation into brain-like endothelial cells

Supplementary Figure 1 Culture, morphology, and marker expression of BM-MSCs. (a–c) Bright-field images of BM-MSCs in LG-DMEM expansion medium, where the whirlpool structure (a and b) and cell bodies crystal formation (c) upon culture continuation are evident. (d) CD-105 expression of BM-MSCs and the differentiated cells. (e) The fluorescence intensity quantified in Fiji software and analyzed in GraphPad Prism. ****p < 0.0001 from 1-way ANOVA comparisons. The scale bars of (a) and (b) are 150 μm, (c) is 80 μm, and (d) is 60 μm.

Supplementary Figure 2 Culture and morphology of differentiated BM-MSCs. (a) Bright-field image of BM-MSCs in IMDM medium on day 4 showing the short spindle. (b) Higher confluency of cells along with culture continuation. (c) Cell body crystals form upon culture continuation on day 9. The scale bars of (a), (b), and (c) are 60 μm, 110 μm, and 80 μm, respectively.

Supplementary Figure 3 Different medium compositions and seeding densities affect differentiation. (a) A schematic timeline for the experiments where BM-MSCs cultured in different differentiation media at (b) 1 × 10^4^, (c) 7.5 × 10^3^, (d) 5 × 10^3^ cells/well in 48 well plates for different incubation periods. (I) ZO-1 and CD-31 expressions tested by immunofluorescence staining. (II) The fluorescence intensity quantified in Fiji software and analyzed in GraphPad Prism. *p < 0.05, **p < 0.01, ***p < 0.001, and ****p < 0.0001 from 2-way ANOVA comparisons. All scale bars are 25 μm.

Supplementary Figure 4 Impact of RA on MSC differentiation into BLECs. The figure shows the quantified (a) ZO-1 and (b) CD-31 expressions after 1μM RA was added for 24 and 48 h on day 6 and day 12 of differentiation. The fluorescence intensity was quantified in Fiji software and analyzed in GraphPad Prism. *p < 0.05, **p < 0.01, and ****p < 0.0001 from 2-way ANOVA comparisons.

Supplementary Figure 5 Testing more marker expressions on the differentiated MSCs. The figure shows the quantified CD-34, occludin, and vWF expression after 3 μM RA was added on day 6 of differentiation for 48 h. The fluorescence intensity was quantified in Fiji software and analyzed in GraphPad Prism. *p < 0.05, **p < 0.01, ***p < 0.001, and ****p < 0.0001 from 2-way ANOVA comparisons.

Supplementary Figure 6 Chemical hypoxia induced by CoCl_2_ affects BM-MSCs differentiation. The figure shows the quantified ZO-1 expression in (a) high without HPC and (b) low with HPC CoCl_2_ concentrations, respectively. The fluorescence intensity was quantified in Fiji software and analyzed in GraphPad Prism. ***p < 0.001 and ****p < 0.0001 from 2-way ANOVA comparisons.

Supplementary Figure 7 MTT assay for CoCl_2_ and Na_2_SO_3_ cytotoxicity. BM-MSCs were cultured in LG-DMEM expansion medium as 2 × 10^3^ cells/well in 96-well plates for (a) 5 and 9 days, (b) 5 and 8 days, and (c) 5 and 9 days with different concentrations: (a) 0.05 μM, 0.2 μM, 1 μM, 5 μM, 25 μM, 50 μM, 100 μM, 200 μM, and 400 μM; (b) 0.005 μM, 0.025 μM, 0.05 μM, 0.1 μM, 0.2 μM, 1 μM, 5 μM, 25 μM, and 50 μM; and (c) 0.025 μM, 0.125 μM, 0.5 μM, 2.5 μM, 10 μM, 50 μM, 200 μM, 1000 μM, and 4000 μM. The figure shows the noncytotoxic safe concentrations of CoCl_2_ and Na_2_SO_3_. The graphs are nonlinear regression curves with logarithmic concentrations.

**Table 1:** Details of differentiation media compositions.

	Complete Medium	Company and Catalog number	Basal Medium	Growth Factors	Company	supplementaiton	Company
**Expansion**	Complete LG-DMEM	Pan-biotech, P04-01515	LG-DMEM			10% hi-FBS	Pan-biotech
						1 % P/S	
						1% 200 mM L-Glu	Merck

	Complete IMDM	Gibco, 12440-053	IMDM			10% hi-FBS	Pan-biotech

						1 % P/S	

**Differentiation**	Complete LG-DMEM	Pan-biotech, P04-01515	LG-DMEM	0.1% VEGF	Lonza, CC-4176	2% FBS	Pan-biotech
				0.4% bFGF			
				0.1% IGF			
				0.1% EGF			
				0.1% Ascorbic Acid			
				0.1% Heparin			
	Complete IMDM	Gibco, 12440-053	IMDM	0.1% VEGF	Lonza, CC-4176	2% FBS	Pan-biotech
				0.4% bFGF			
				0.1% IGF			
				0.1% EGF			
				0.1% Ascorbic Acid			
				0.1% Heparin			
	Complete Endopan	Pan-biotech, P04-0065K	Endopan 300 SL	0.1% hr-VEGF	Pan-biotech, P04-0065K	2% FBS	Pan-biotech
				0.1% hr-bFGF-2			
				0.1% R3-IGF-1			
				0.1% hr-EGF			
				0.1% Ascorbic Acid			
				0.1% Heparin			
				0.02% Hydrocortisone			
				0.06% G/A			
	Complete EGM-2	Lonza, CC-4176	EBM-2	0.1% VEGF	Lonza, CC-4176	2% FBS	Lonza
				0.4% hFGF-B			
				0.1% R3-IGF-1			
				0.1% h-EGF			
				0.1% Ascorbic Acid			
				0.1% Heparin			
				0.04% Hydrocortisone			
				0.1% G/A			
LG-DMEM		Low-glucose Dulbecco’s Modified Eagle Medium					
IMDM		Iscove’s Modified Dulbecco Medium					
EBM-2		Endothelial basal medium					
VEGF		Vasular endothelial growth factor					
bFGF		basic Fibroblast growth factor					
IGF		Insulin-like growth factor					
EGF		Epidermal growth factor					
G/A		gentamycin/amphotericin					
hi-FBS		heat-inactivated fetal bovine serum					
FBS		fetal bovine serum					
P/S		Penicillin-Streptomycin					
L-Glu		L-glutamine					

## Data Availability

All data generated or analyzed during this study are included in this published article or are available from the corresponding author upon request.
