# Supplementary figures and images for: Human bone marrow-mesenchymal stem cells differentiation into brain-like endothelial cells
Source: Turk J Biol. 2025 Aug 5;50(1):1–16. doi: 10.55730/1300-0152.2786 (PMC12978769; doi:10.55730/1300-0152.2786)

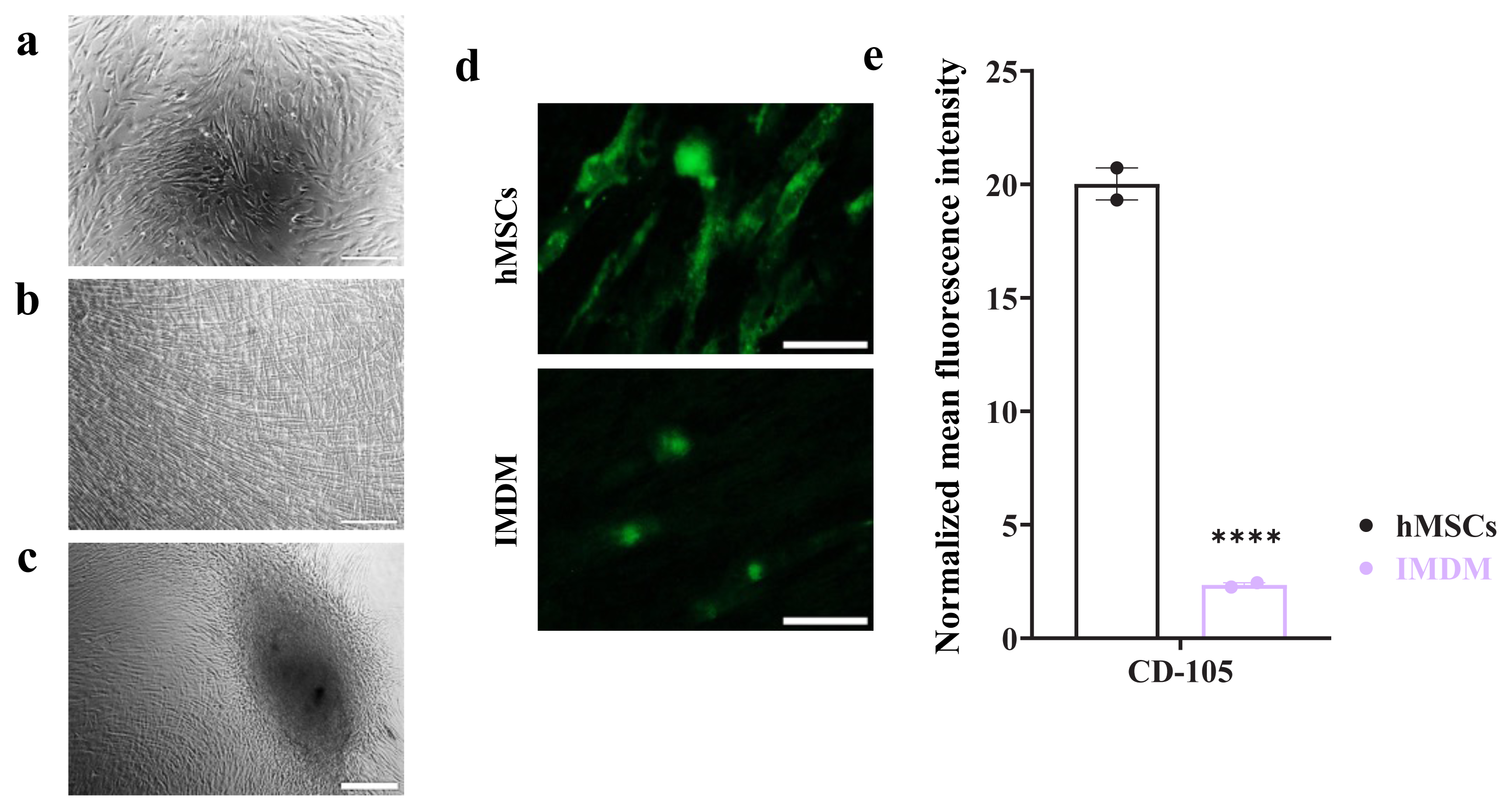

Supplement: Supplementary Figure 1 — Culture, morphology, and marker expression of BM-MSCs. (a–c) Bright-field images of BM-MSCs in LG-DMEM expansion medium, where the whirlpool structure (a and b) and cell bodies crystal formation (c) upon culture continuation are evident. (d) CD-105 expression of BM-MSCs and the differentiated cells. (e) The fluorescence intensity quantified in Fiji software and analyzed in GraphPad Prism. ****p < 0.0001 from 1-way ANOVA comparisons. The scale bars of (a) and (b) are 150 μm, (c) is 80 μm, and (d) is 60 μm. [file tjb-50-01-1s1.tif]

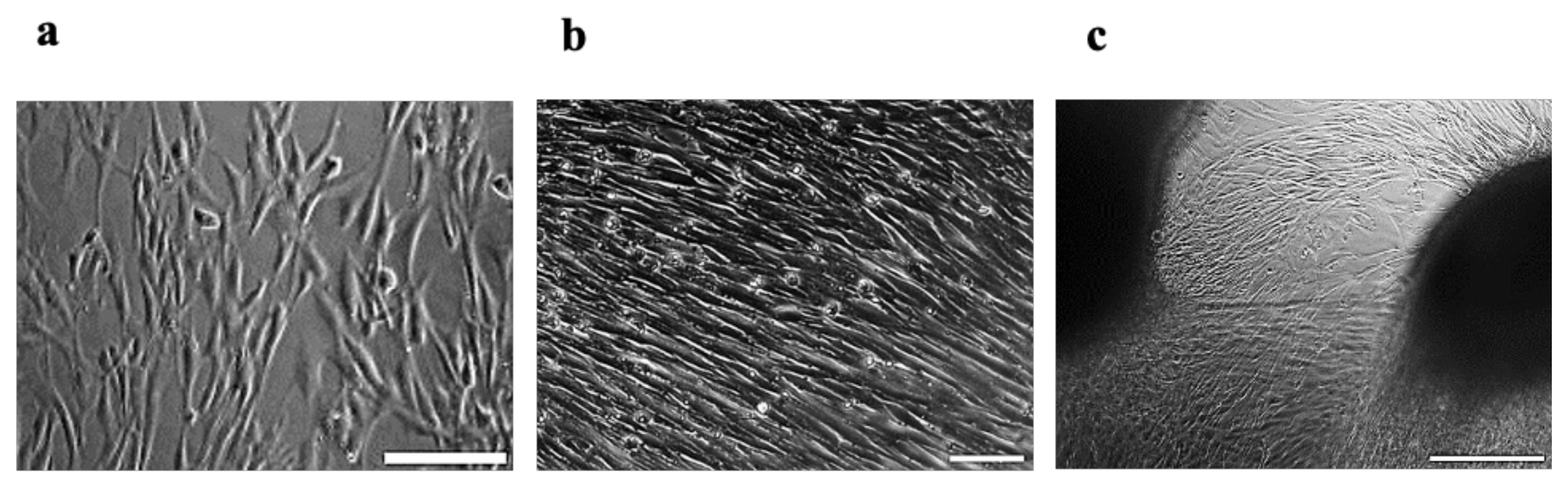

Supplement: Supplementary Figure 2 — Culture and morphology of differentiated BM-MSCs. (a) Bright-field image of BM-MSCs in IMDM medium on day 4 showing the short spindle. (b) Higher confluency of cells along with culture continuation. (c) Cell body crystals form upon culture continuation on day 9. The scale bars of (a), (b), and (c) are 60 μm, 110 μm, and 80 μm, respectively. [file tjb-50-01-1s2.tif]

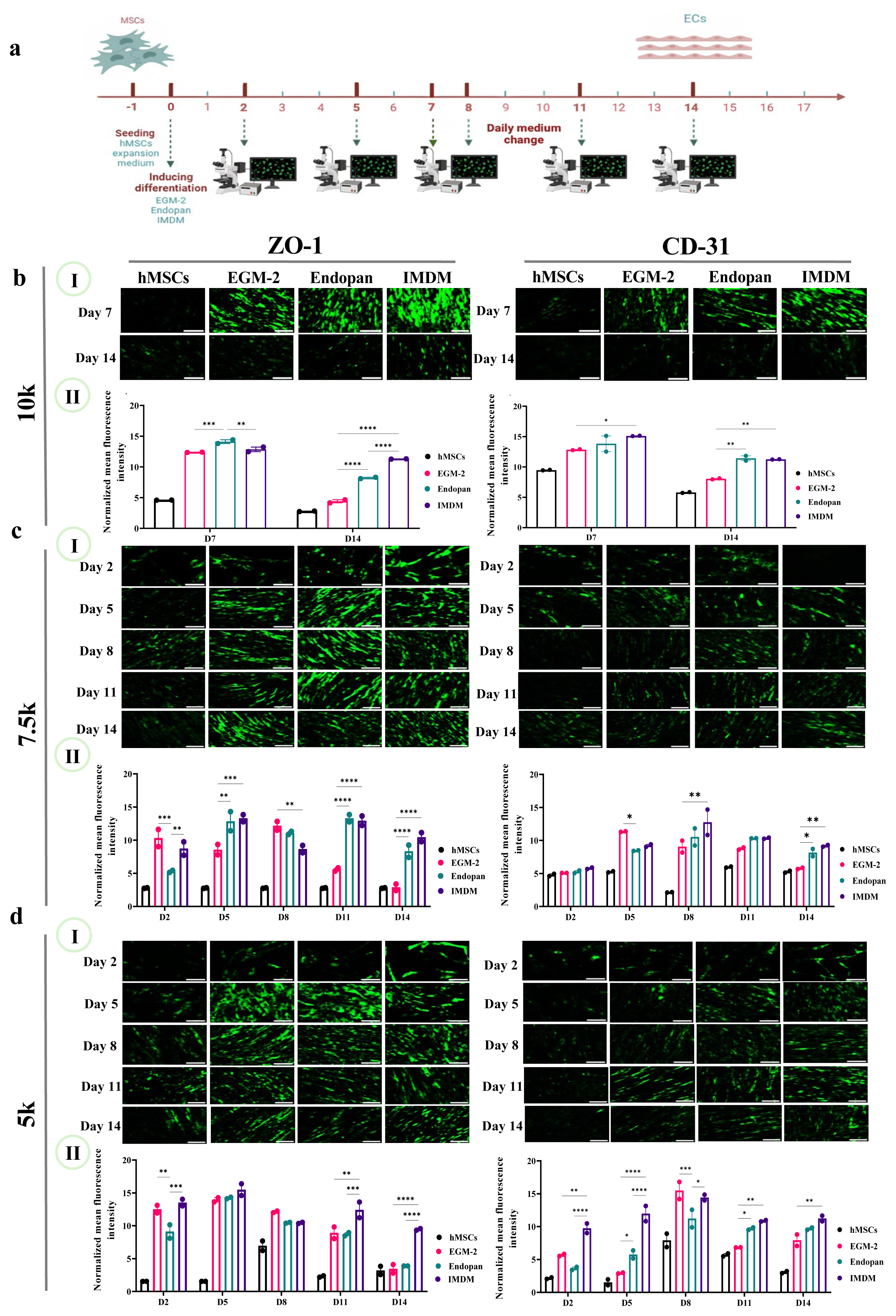

Supplement: Supplementary Figure 3 — Different medium compositions and seeding densities affect differentiation. (a) A schematic timeline for the experiments where BM-MSCs cultured in different differentiation media at (b) 1 × 10^4^, (c) 7.5 × 10^3^, (d) 5 × 10^3^ cells/well in 48 well plates for different incubation periods. (I) ZO-1 and CD-31 expressions tested by immunofluorescence staining. (II) The fluorescence intensity quantified in Fiji software and analyzed in GraphPad Prism. *p < 0.05, **p < 0.01, ***p < 0.001, and ****p < 0.0001 from 2-way ANOVA comparisons. All scale bars are 25 μm. [file tjb-50-01-1s3.tif]

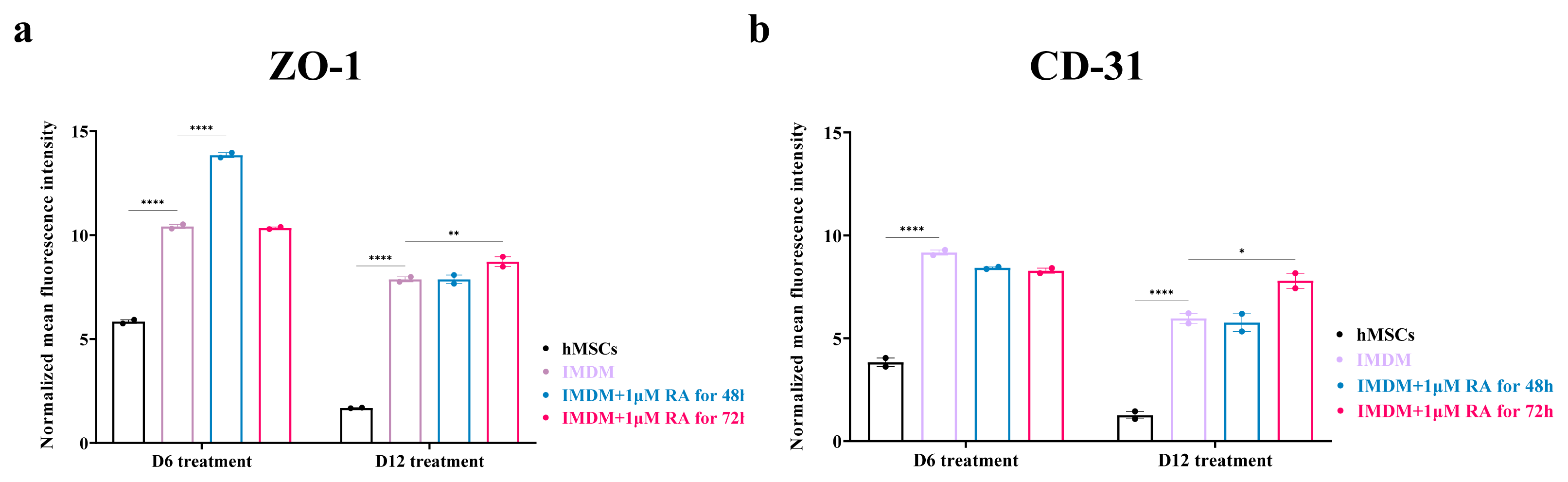

Supplement: Supplementary Figure 4 — Impact of RA on MSC differentiation into BLECs. The figure shows the quantified (a) ZO-1 and (b) CD-31 expressions after 1μM RA was added for 24 and 48 h on day 6 and day 12 of differentiation. The fluorescence intensity was quantified in Fiji software and analyzed in GraphPad Prism. *p < 0.05, **p < 0.01, and ****p < 0.0001 from 2-way ANOVA comparisons. [file tjb-50-01-1s4.tif]

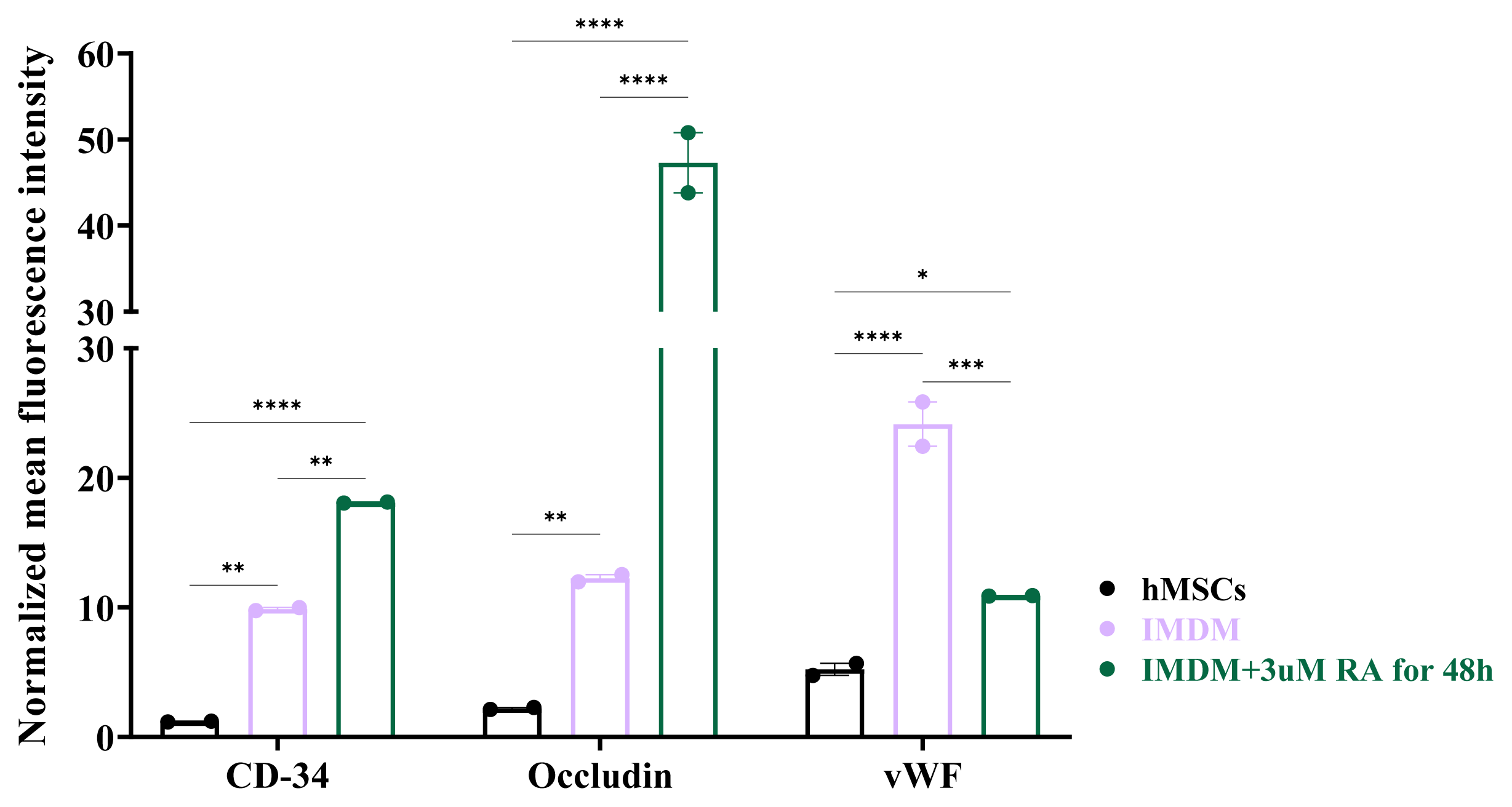

Supplement: Supplementary Figure 5 — Testing more marker expressions on the differentiated MSCs. The figure shows the quantified CD-34, occludin, and vWF expression after 3 μM RA was added on day 6 of differentiation for 48 h. The fluorescence intensity was quantified in Fiji software and analyzed in GraphPad Prism. *p < 0.05, **p < 0.01, ***p < 0.001, and ****p < 0.0001 from 2-way ANOVA comparisons. [file tjb-50-01-1s5.tif]

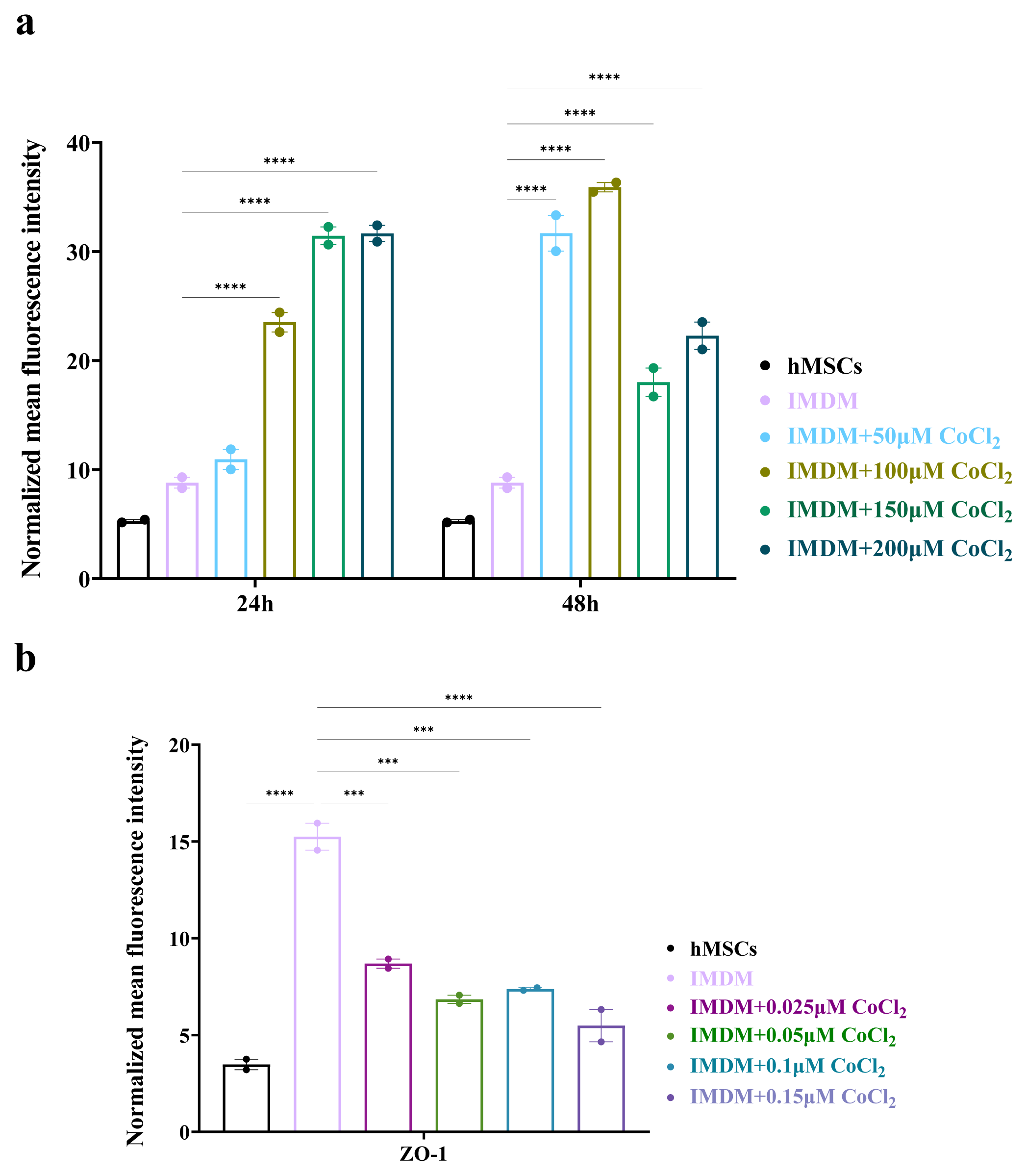

Supplement: Supplementary Figure 6 — Chemical hypoxia induced by CoCl2 affects BM-MSCs differentiation. The figure shows the quantified ZO-1 expression in (a) high without HPC and (b) low with HPC CoCl2 concentrations, respectively. The fluorescence intensity was quantified in Fiji software and analyzed in GraphPad Prism. ***p < 0.001 and ****p < 0.0001 from 2-way ANOVA comparisons. [file tjb-50-01-1s6.tif]

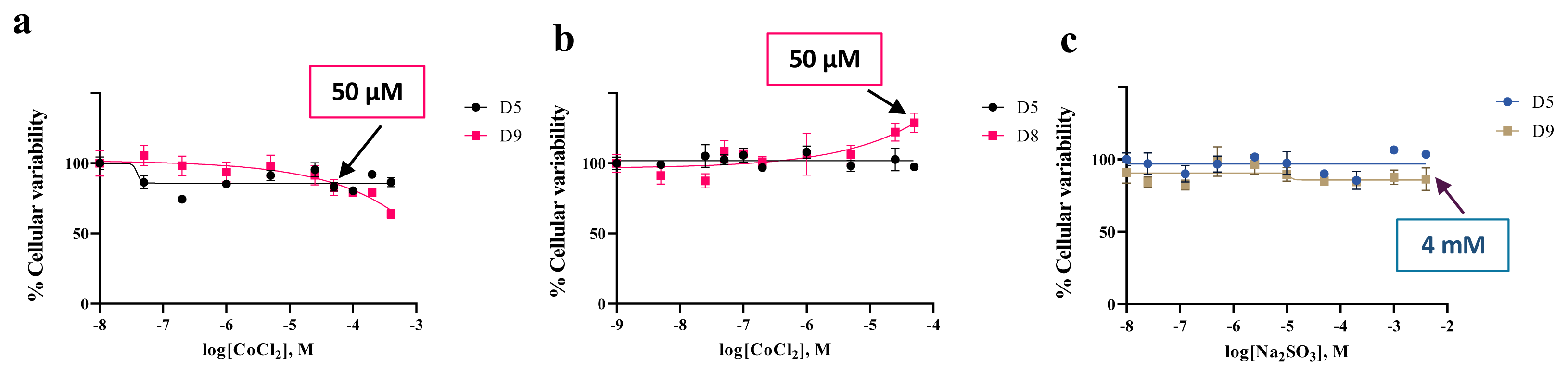

Supplement: Supplementary Figure 7 — MTT assay for CoCl2 and Na2SO3 cytotoxicity. BM-MSCs were cultured in LG-DMEM expansion medium as 2 × 10^3^ cells/well in 96-well plates for (a) 5 and 9 days, (b) 5 and 8 days, and (c) 5 and 9 days with different concentrations: (a) 0.05 μM, 0.2 μM, 1 μM, 5 μM, 25 μM, 50 μM, 100 μM, 200 μM, and 400 μM; (b) 0.005 μM, 0.025 μM, 0.05 μM, 0.1 μM, 0.2 μM, 1 μM, 5 μM, 25 μM, and 50 μM; and (c) 0.025 μM, 0.125 μM, 0.5 μM, 2.5 μM, 10 μM, 50 μM, 200 μM, 1000 μM, and 4000 μM. The figure shows the noncytotoxic safe concentrations of CoCl2 and Na2SO3. The graphs are nonlinear regression curves with logarithmic concentrations. [file tjb-50-01-1s7.tif]
